# Cutting-Edge Technology for Rapid Bedside Assessment of Capillary Refill Time for Early Diagnosis and Resuscitation of Sepsis

**DOI:** 10.3389/fmed.2020.612303

**Published:** 2020-12-21

**Authors:** David C. Sheridan, Robert Cloutier, Andrew Kibler, Matthew L. Hansen

**Affiliations:** ^1^Department of Emergency Medicine, Oregon Health and Science University, Portland, OR, United States; ^2^Promedix Inc., Portland, OR, United States

**Keywords:** capillary refill, objective, device, sepsis 2, emergency care

## Abstract

Sepsis currently affects over 30 million people globally with a mortality rate of ~30%. Prompt Emergency Department diagnosis and initiation of resuscitation improves outcomes; data has found an 8% increase in mortality for every hour delay in diagnosis. Once sepsis is recognized, the current Surviving Sepsis Guidelines for adult patients mandate the initiation of antibiotics within 3 h of emergency department triage as well as 30 milliliters per kilogram of intravenous fluids. While these are important parameters to follow, many emergency departments fail to meet these goals for a variety of reasons including turnaround on blood tests such as the serum lactate that may be delayed or require expensive laboratory equipment. However, patients routinely have vital signs assessed and measured in triage within 30 min of presentation. This creates a unique opportunity for implementation point for cutting-edge technology to significantly reduce the time to diagnosis of potentially septic patients allowing for earlier initiation of treatment. In addition to the practical and clinical difficulties with early diagnosis of sepsis, recent clinical trials have shown higher morbidity and mortality when septic patients are over-resuscitated. Technology allowing more real time monitoring of a patient's physiologic responses to resuscitation may allow for more individualized care in emergency department and critical care settings. One such measure at the bedside is capillary refill. This has shown favor in the ability to differentiate subsets of patients who may or may not need resuscitation and interpreting blood values more accurately (1, 2). This is a well-recognized measure of distal perfusion that has been correlated to sepsis outcomes. This physical exam finding is performed routinely, however, there is significant variability in the measurement based on who is performing it. Therefore, technology allowing rapid, objective, non-invasive measurement of capillary refill could improve sepsis recognition compared to algorithms that require lab tests included lactate or white blood count. This manuscript will discuss the broad application of capillary refill to resuscitation care and sepsis in particular for adult patients but much can be applied to pediatrics as well. The authors will then introduce a new technology that has been developed through a problem-based innovation approach to allow clinicians rapid assessment of end-organ perfusion at the bedside or emergency department triage and be incorporated into the electronic medical record. Future applications for identifying patient decompensation in the prehospital and home environment will also be discussed. This new technology has 3 significant advantages: [1] the use of reflected light technology for capillary refill assessment to provide deeper tissue penetration with less signal-to-noise ratio than transmitted infrared light, [2] the ability to significantly improve clinical outcomes without large changes to clinical workflow or provider practice, and [3] it can be used by individuals with minimal training and even in low resource settings to increase the utility of this technology. It should be noted that this perspective focuses on the utility of capillary refill for sepsis care, but it could be considered the next standard of care vital sign for assessment of end-organ perfusion. The ultimate goal for this sensor is to integrate it into existing monitors within the healthcare system.

## Introduction

Sepsis is the leading cause of death in United States (US) hospitals (3, 4). Globally, sepsis affects 30 million people annually including 3 million children with a mortality rate of ~30% (5–7). Furthermore, sepsis is the number 1 cause of both hospitalization and readmission in the U.S. with and approximate annual cost of $27 billion and $2 billion respectively (8). With sepsis currently affecting more than 1.7 million individuals in the US, technological advances to improve diagnosis and monitoring could save many lives (9). One particular area that can have a substantial impact is improved recognition in the early phases of sepsis as every hour delay in the diagnosis and treatment increases mortality by 8%.

Once sepsis has been detected it is key to resuscitate patients in a timely fashion. The current surviving sepsis guidelines have recommended fluid administration (30 mls per kilogram) to all patients within the first 3 h (10). This approach is suggested based on sepsis related decreases in end-organ perfusion with fluids helping optimize oxygen delivery. However recent studies have challenged this recommendation showing that over-resuscitation may in fact increase mortality (11). Therefore, the ability to individualize therapy and direct resuscitation at the bedside in real-time based on the patient physiology and response to therapeutic interventions is vital. The current standard of care focuses on following the surviving sepsis campaign recommendations and relies heavily on blood tests for end-organ function/perfusion; however, there may be better alternatives. The sepsis-3 definitions have shown more focus on clinical variables that can be obtained quickly and non-invasively which an additional measure like capillary refill could improve upon (12). One large clinical trial enrolled and randomized septic patients into two resuscitation arms; one whose treatment was managed via serial blood lactate levels or, the other, via serial capillary refill assessment (13, 14). If a patient did not have normalization of their capillary refill time or serum lactate they were given more fluids. Capillary refill-guided resuscitation demonstrated more favorable outcomes in terms of morbidity and mortality than the current standard, serum lactate.

Capillary refill is not a novel data point, it is well-known to medical providers and is taught routinely in nursing and medical schools. It is a physical finding that should be assessed and documented on every acutely ill patient as it is a marker of distal perfusion (15). The correlation between organ perfusion and peripherally measured capillary refill time has been well-studied (16, 17). One study of sepsis patients created a protocol to withhold further intravenous fluids in patients that had normalized their capillary refill times and found decreased end organ failure compared to those in the standard care arm (18). A follow up study compared therapeutic monitoring via capillary refill to metabolic parameters, including central venous oxygen saturation and found the presence of normalized capillary refill time at 6 h was independently and significantly associated with successful resuscitation (19). Recent trials have successively demonstrated the ability of capillary refill to dynamically reflect physiologic responses to fluid challenges in patients with various types of shock (20). Consequently, intensive care unit protocols are being developed with the aim of normalizing capillary refill as a guide to more targeted and individualized sepsis care (21). The difficulty to date with capillary refill is its subjective nature. Our group performed a study evaluating capillary refill in healthy subjects by board certified physicians with video and found statistically significant variability within and between providers (22).

Emergency and Critical Care physicians are key to the improved care of sepsis. One way to accomplish this is through innovation and fostering technology development within our specialty, capitalizing on the experience of bedside clinicians, rather than relying on industry. This type of work is critical to improve healthcare, and emergency physicians should be leaders in this area as we deliver care across multiple and varied care settings. Based on a recent publication in the American Journal of Emergency Medicine, our group followed a problem-based innovation approach to develop a new bedside technology for sepsis and other conditions to more effectively and reliably monitor distal tissue perfusion (23). This approach identifies a clinically relevant problem in the emergency department and then works to develop a solution. By defining the problem very well and specific the solution can be developed more robustly.

## Cutting-Edge Technology

Flowsense is a capillary refill measurement system designed with clinical users in mind. This device was designed to optimize ease of use and data collection quality based on an innovation feedback cycle from two previous design and data collection study generations performed by our team. It is a wireless portable finger sensor with a streamlined application that guides users through the measurement process, and provides real-time feedback on measurement quality, requesting repeated measure when necessary. These features ensure reproducible, reliable data for timely critical interventions and medical decision making.

The Flowsense finger sensor is a battery-operated optical, temperature, and force measurement instrument which collects highly accurate real-time signals, provides user input and feedback through an operation button and multicolor LED, and allows wipe-down disinfection in between uses. This unit combines a highly integrated, lightweight and compact finger-mounted sensor stack with a wrist-mounted wireless transceiver and user control. This design minimizes motion and acceleration artifact at the finger during data collection, and provides a comfortable watch-like wrist mount for digital signal processing and control interface. The system workflow allows for simple initiation at the application level and 100% remote user operation of the wireless finger sensor without requiring app interaction while taking measurements.

**Figure 1:**
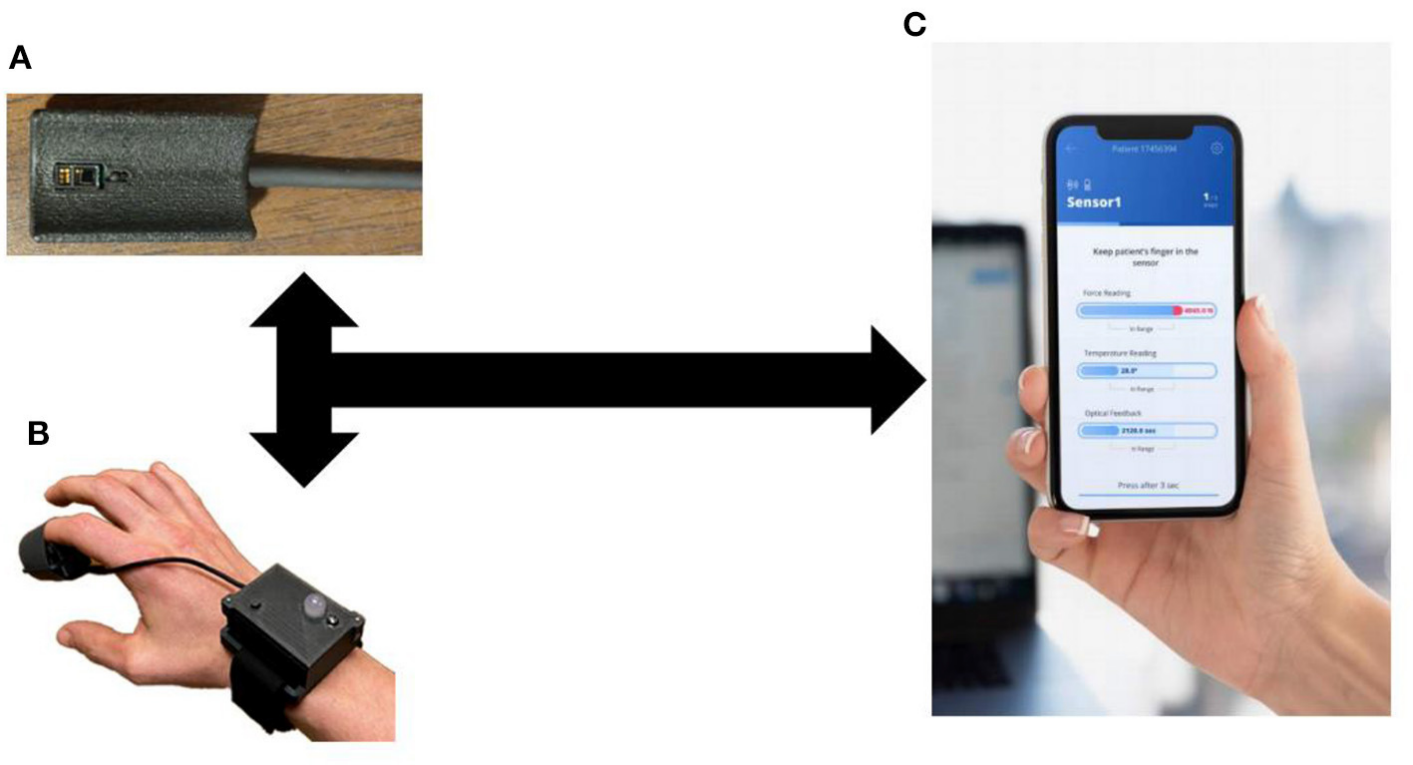
Capillary Refill Technology. **(A)** Finger Sensor; **(B)** Sensor on a patient; **(C)** Mobile application to process real-time data collection.

Upon measurement completion, final results and data graphs are displayed to the user, and data is uploaded to a secure cloud server automatically for post-processing (Figure 1). This cloud-based platform will enable system integration with digital health records, physician user alerts, and a continuous process of capability improvement through enhanced clinical feature extraction enabled by machine learning and artificial intelligence techniques. A pilot cohort of patients in the emergency department were enrolled to compare the signal processing algorithms from this device to manual assessment of capillary refill (24). This study found that the algorithm for capillary refill assessment showed a good correlation to expert trained capillary refill assessment (Pearson coefficient 0.7).

### Technical Detail

The current design focuses on a stand-alone technology for data collection. As can be seen from Figure 1, this technology can integrate into current medical monitors and be used routinely during vital sign assessment. In addition, the stand-alone device allows its use in the prehospital, outpatient or home environment.

Each finger sensor is calibrated and uniquely serialized, and has the ability to pair with any approved control unit. As such, pairing flexibility and system swap capability is maintained to ensure high clinical throughput and low down-time in case finger sensor or battery replacement is required. Together, these features make the Flowsense system the only system capable of accurately measuring capillary refill, with a design oriented toward clinical use and low electronics parts cost (~$15), enabling future home use as well. Prior reports and research have examined methods to assess capillary refill mainly with the use of standard pulse oximetry waveforms (25, 26). This has shown significant promise for capillary refill in its ability to correlate with blood lactate levels and detect sepsis earlier while monitoring critically ill adult patients (27, 28). The technology described in this report is unique and may be superior to prior technology in that it does not use transmitted pulse oximetry. Instead as described it utilizes reflected light with the ability to penetrate deeper into the capillary beds than infrared light with less signal-to-noise ratio. The pressure application is through a simple manual method rather than an expensive pneumatic bladder. To increase the reproducibility, the accompanying application instructs the user to apply pressure for 3 s keeping it within a steady range. If the application senses a significant deviation, including too high or low of pressure, or that pressure was not rapidly released it will notify the user and help them troubleshoot. This expands the utility of the technology to providers with limited training to more experience.

## Economic Model

As discussed earlier, sepsis care is costly to hospital systems (8). To further investigate the economic impact a technology to rapidly and accurately measure capillary refill could have, specifically in the emergency department, our group worked with a large academic medical center in the United States. This medical center cared for 2,606 patients with the final primary diagnosis of sepsis over a 2-year period (July 2018 through July 2020); we were able to obtain financial data on 1,571 patients. This data included revenue to the hospital, charges, and cost data calculated as direct and indirect costs. The operating margin was calculated as the difference in the revenue generated minus the direct/indirect costs the hospital incurred to provide the care. Over this time frame in the care for the ~1,600 patients, hospital revenue was $82,726,206.93 while incurring $86,887,008.45 in costs. This resulted in a net ***negative***operating margin of ~$4,200,000 for sepsis care.

**Figure 2:**
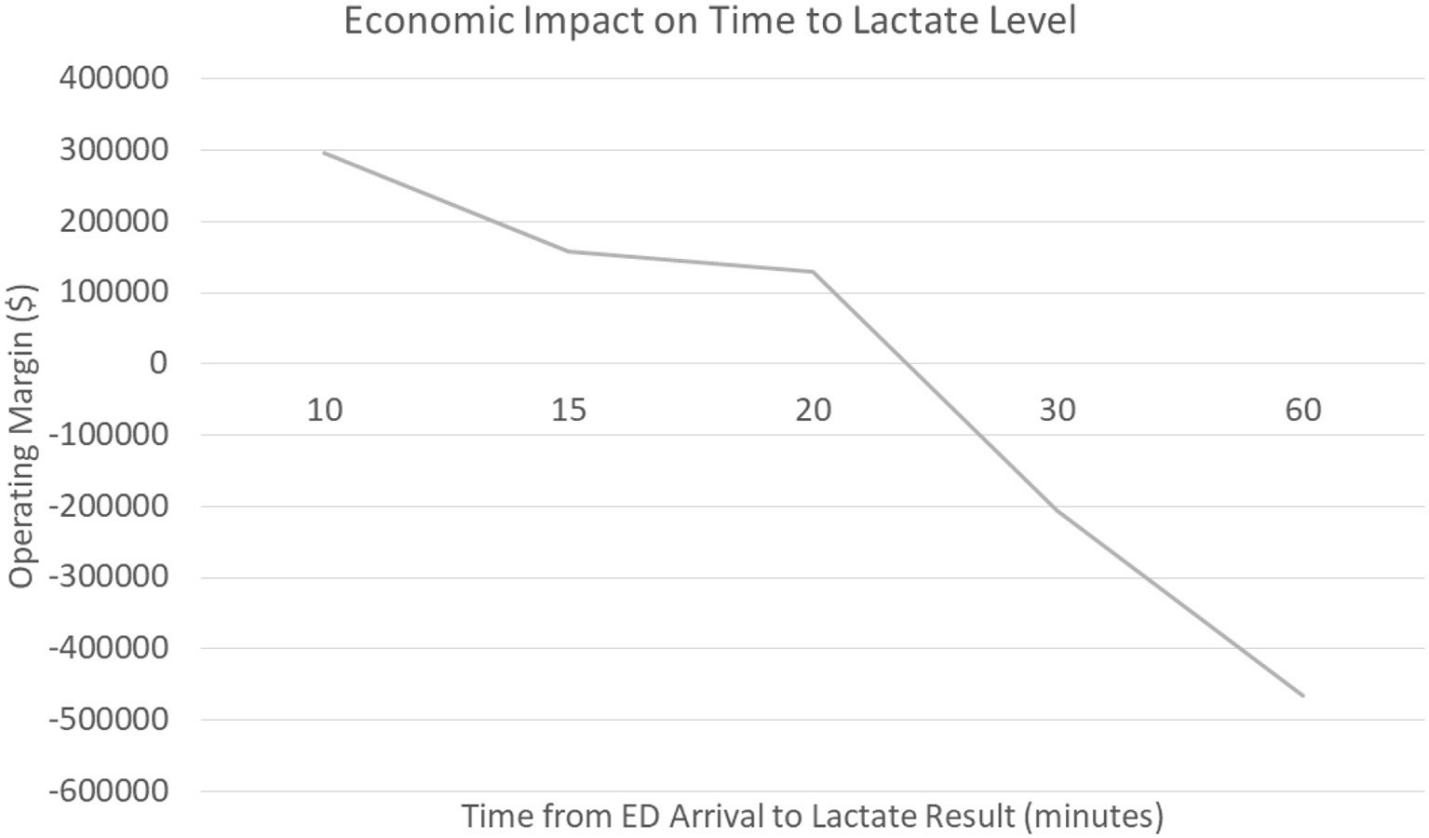
Economic Impact of Emergency Department Sepsis Recognition.

We hypothesize that delayed recognition in the emergency department could be a significant contributor to the negative margin associated with sepsis care. The data shows that patients who had a serum lactate drawn within the first 10 min generated a *positive* operating margin for the hospital (+$296,000) compared to patients who had their serum lactate within 60 min who generated a *negative* operating margin (-$466,000). Furthermore, when evaluating patients who presented to the emergency department at this medical center vs. direct admits, the data shows a significant trend that delays in serum lactate levels resulted in significant costs to the hospital; *an opportunity that non-invasive technology may improve* (Figure 2) The average time for drawing blood tests (lactate) related to sepsis diagnosis in the emergency department was ~61 min, though non-invasive vital signs were performed on average within 8 min of emergency department arrival; a simple technology to measure capillary refill could be incorporated with existing monitors and be obtained in a very timely fashion. Our voice of customer work with providers showed the preference and ability to measure capillary refill time using our technology along with other routine vital signs. Therefore, our technology has the ability to be used during initial emergency department triage, within the first 10 min, and offers a significant opportunity to improve healthcare costs in addition to patient outcomes.

## Discussion

Through a problem-based approach, our group was able to develop a new technology for assessment of distal perfusion in the care of sepsis (23). Sepsis results in significant end-organ dysfunction due to distributive shock that shunts blood away from the capillary bed in the finger to preserve oxygenated blood for vital organs (29). Capillary refill assessment is very subjective and literature has shown that under ideal circumstances physicians have high variability in their assessment (22). Our technology increases the ability of personnel, with both advanced and limited training, to monitor patients in a variety of settings ranging from the ED, the intensive care unit, the prehospital setting and potentially even the home. The current device is primed for clinical research and undergoing testing in the both the ICU and ED setting to further validate its clinical evidence.

## Author Contributions

DS, AK, and MH developed the technology. DS prepared the first draft, critically revised, and approved final draft of manuscript. RC, MH, and AK critical reviewed and approved the final draft of the manuscript. All authors contributed to the article and approved the submitted version.

## Conflict of Interest

DS, MH, and AK have an equity interest in Promedix Inc. which is developing a capillary refill technology. The remaining author declare that the research was conducted in the absence of any commercial or financial relationships that could be construed as a potential conflict of interest.
